# Inhibition of long noncoding RNA cancer susceptibility candidate 7 attenuates hepatocellular carcinoma development by targeting microRNA-30a-5p

**DOI:** 10.1080/21655979.2022.2068289

**Published:** 2022-04-29

**Authors:** Dongsheng Li, Lin Lu, Miaomiao Liu, Jufeng Sun

**Affiliations:** Hepatobiliary Surgery, The First Affiliated Hospital of Jinzhou Medical University, Jinzhou, Liaoning, China

**Keywords:** CASC7, miR-30a-5p, KLF10/TGF-β/SMAD3, hepatocellular carcinoma, proliferation

## Abstract

Long non-coding RNA (lncRNA) cancer susceptibility candidate 7 (CASC7) was reported to be participated in tumor development. This study was carried out to investigate the functions of CASC7 in hepatocellular carcinoma (HCC) progression. The expression of CASC7 and microRNA-30a-5p (miR-30a-5p) in HCC tissues and cells were detected by quantitative Real-time PCR (qRT-PCR). The expression of Krueppel-like factor 10 (KLF10), transforming growth factor-β (TGF-β), and SMAD3 were detected by Western Blot analysis. Transwell assay, flow cytometry, Cell Counting Kit-8 (CCK-8) assay and colony formation assay were performed to evaluate the effects of CASC7, KLF10 and miR-30a-5p on cell function. The relationship among CASC7, KLF10 and miR-30a-5p was evaluated by luciferase reporter assay and bioinformatics analyses. Tumor growth was detected in nude mice. The expression levels of CASC7 were increased and the expression levels of miR-30a-5p were reduced in HCC cells and tissues. Knockdown of CASC7 and overexpression of miR-30a-5p reduced tumor growth as well as HCC cell proliferation, invasion and migration. In HCC tumor tissues, the expression of miR-30a-5p was negatively correlated with the expression of CASC7. Moreover, as a target of miR-30a-5p, KLF10 was regulated by CASC7 and miR-30a-5p, and CASC7 regulated the KLF10/TGF-β/SMAD3 pathway via binding to miR-30a-5p, thereby promoting HCC cell progression.

## Graphical Abstract

**Figure:**
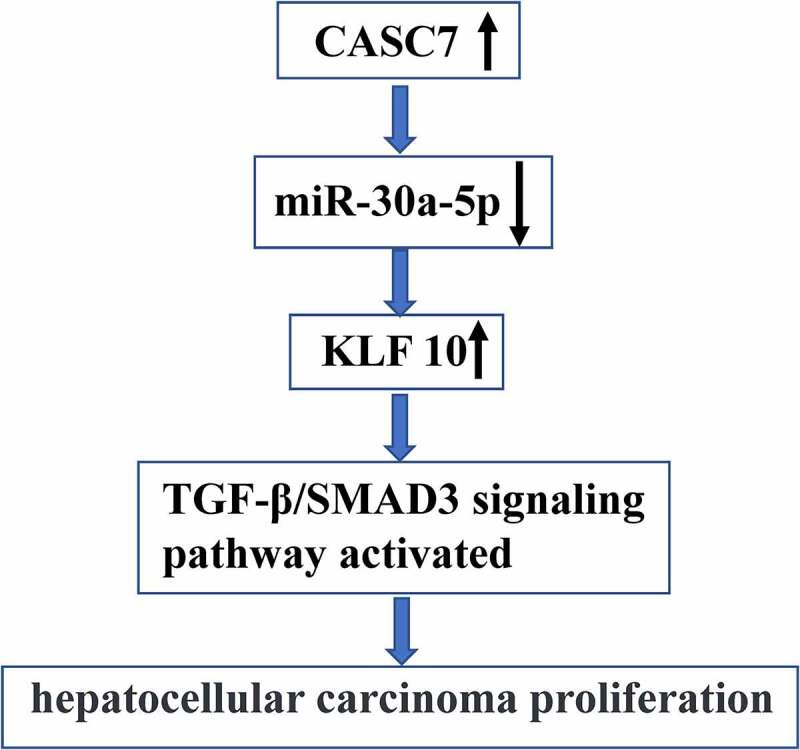

## 1. Introduction

HCC has become the sixth most common malignant tumor in the world [1,2], and most of them are diagnosed in late stages with multiple complications and the surgical resection rate is extremely low [3]. At present, the treatment for liver cancer is mainly surgical resection, combined with radiotherapy, chemotherapy and liver transplantation [4]. Most patients often suffer from metastasis and recurrence after surgery [5]. Recent studies have shown that HCC is closely related to abnormal expression of long non-coding RNAs (lncRNAs) and cell signal transductions [6].

LncRNAs are expressed in different cells and tissues and can interact with other macromolecules such as DNAs, miRNAs and proteins [7,8]. With the advance in high-throughput sequencing technologies, various lncRNAs have been identified [9]. Moreover, lncRNAs might serve as the biomarkers for tumor progression and recurrence in HCC patients [10,11]. For example, it has been reported that lncRNA MIAT promotes HCC cell invasion and proliferation via sponging miR-214 [12]. LncRNA cancer susceptibility candidate 7 (CASC7) was revealed to be involved in glioma [13], non-small cell lung cancer [14], and colon cancer [15]. However, the functions of CASC7 in HCC remain unclear.

MicroRNAs (miRNAs) widely exist in many organisms and are involved in the regulation of gene expression [16]. Studies have found that lncRNAs can regulate the expression of miRNAs and tumorigenesis [17]. Moreover, miRNAs are also involved in liver cancer [18] and function as oncogenes or tumor suppressive genes [19]. Meanwhile, miRNAs are related to the clinicopathological characteristics of liver cancer such as pathological type and the degree of malignancy [20]. As a newly discovered miRNA, miR-30a-5p has been reported to be involved in invasion and migration of a variety of cancers [21]. Moreover, studies have found that miR-30a-5p is significantly downregulated and suppresses HCC tumor growth and cell invasion and proliferation in HCC tissues [22]. We therefore speculated that miR-30a-5p may mediate the function of CASC7 in HCC. The TGF-β/SMAD3 signaling participates in HCC migration and metastasis [15,23]. In addition, it has been reported that miRNA-491 is involved in anti-angiogenesis in HCC induced by arsenic trioxide by inhibiting the TGF-β/SMAD3 signaling [14].

Furthermore, the TGF-β/SMAD3 pathway mediated the anti-oncogene of miR-133a/FOSL2 in HCC. These studies suggest that the TGF-β/SMAD3 signaling can be regulated by miRNAs in HCC. We therefore hypothesized that CASC7 might regulate the KLF10/TGF-β/SMAD3 pathway through miR-30a-5p in HCC development. This study was therefore carried out to investigate the role and underlying mechanism of CASC7 in HCC.

## 2. Methods

### 2.1. Clinical specimens

The tumor tissues and adjacent non-tumor tissues were obtained from 38 patients who were admitted at the First Affiliated Hospital of Jinzhou Medical University between January 2012 to July 2014. All patients signed the informed consent. Among the 38 patients, 20 patients developed intrahepatic metastases. During 2 years, 18 patients had solitary tumors and no metastasis or recurrence occurred. This study was approved by the Ethics Committee of the aforementioned hospital. The Correlation between clinicopathologic characteristics and the expression of CASC7 was analyzed in HCC (Table 1).

**Table 1.** Correlation between the clinicopathologic characteristics and CASC7 expression in HCC (n = 50)

Clinical parameters	Cases	Expression level		*P* value
		CASC7^high^ (*n* = 28)	CASC7^low^ (*n* = 22)	
Age (years)				
<50	20	12	8	0.338
>50	30	16	14	
Gender				
Male	36	20	16	0.0311
Female	14	8	6	
HBV				
Absent	13	8	5	0.0148
Present	37	20	17	
Serum AFP(ng/ml)				
<400	17	12	5	0.182
>400	33	16	17	
Tumor size (cm)				
<5	28	16	12	0.314
>5	22	12	10	
Number of tumor nodules				
1	35	19	16	0.138
>2	15	9	6	
Cirrhosis				
Absent	15	8	7	0.0991
Present	35	20	15	
Venous infiltration				
Absent	36	20	16	0.282
Present	14	8	6	
Edmondson-Steiner grading				
I+ II	36	22	14	0.331
III+IV	14	6	8	
TNM tumor stage				
I+ II	40	24	16	0.116
III+IV	10	4	6	

*HCC* hepatocellular carcinoma, *HBV* hepatitis B virus, *AFP* alpha-fetoprotein, *TNM* tumor-node-metastasis

### 2.2. Cell lines and cell culture

HepG2, MHCC97H, LO2, HepG2, SK-Hep1, HUH7 and HCCLM3 cell lines were obtained from Tongpai Technology (Shanghai, China). Cells were incubated in DMEM medium with FBS (10%) at 37°C with 5% CO_2_.

### 2.3. Cell transfection

ShCASC7, miR-30a-5p inhibitors and mimic and their controls were purchased from Shanghai Gene Pharmaceutical Co., Ltd. The sequences were as follows: shCASC7-1: 5'-TGGAACACATGGTCCA GCACTTTAA-3'; shCASC7-2: 5'-CATCTATT GTCGTGTTTAAGCTT-3'; shNC: 5’-TGGACAC TGGTGACCTCACTAATAA-3'.Lipofectamine 2000 (Invitrogen, Carlsbad, CA) mediated transfection of oligonucleotides into cells was then performed.

### 2.4. CCK8 assay

Cells (50,000 cells per well) were seeded, and CCK8 solution (100 μL, Liji, Shanghai, China) was added to incubate the cells. Then, the absorbance was measured at 450 nm using a microplate reader (Meigu, Shanghai, China) [24].

### 2.5. RNA pull-down assay

Cells were transfected with biotin-labeled mutant (Mut)- and the wild-type (WT)-bio-miR-30a-5p (50 nM) for 48 h. After washing with PBS, a specific lysis buffer was added to incubate the cells for 10 min. After pre-coating with BSA, M-280 streptavidin magnetic beads were used to incubate the lysate at 4°C for 3 h. Finally, The binding RNA was purified and its concentration was measured [25].

### 2.6. Apoptosis assay

Cells (5 x 10^5^ cells/well) were plated and grown to the logarithmic growth phase. Cells were then collected, counted and separated by centrifugation and regenerated by adding 195 μl annexin V-FITC. Propidium iodide (10 μl) and Annexin V-FITC (5 μl) staining solutions were then added. Cells were incubated in darkness for 10–20 min and then placed in an ice bath [26]. Flow cytometry results were then collected (Jiyuan, Guangzhou, China).

### 2.7. Luciferase reporter assay

Luciferase reporter assay was carried out as previously described [27]. The WT and MUT primers were used to amplify CASC7. pGL3-Bashc luciferase reporter vector carrying the CASC7 fragment was named as CASC7-WT-Luc. The mutant plasmid CASC7-Mut-Luc was constructed by mutagenizing the binding region in CASC7 and miR-30a-5p. pRLTK and miR-30a-5p mimic were co-transfected into cells with CASC7-Mut-Luc and CASC7-WT-Luc. Cells were collected after 6 h of transfection. Luciferase activity was determined using a Luciferase Assay Kit.

### 2.8. RNA extraction and RT-qPCR

otal RNAs were extracted using TRIzol reagent (Aosentai, Shanghai, China). RT-qPCR was carried out as previously described [27]. The quality of RNA samples was analyzed by NanoDrop 1000. cDNA synthesis and RT-qPCR were carried out using qScript microRNA cDNA synthesis kit and ABI 7,500 real-time PCR system, respectively. The expression levels of CASC7 and miR-30a-5p were calculated using the 2^−ΔΔCT^ method. Gene expression levels were normalized to the internal reference U6 and GADPH. The primer sequences were: U6, 5'-CGCTTCACGAATTTGCGTGTCA-3' (forward) and 5'-GCTTCGGCAGCACATATACTAAAAT-3' (reverse); CASC7, 5'-ATCAACGTCAAGCTGGGA GG-3' (forward) and 5'-CTTGTCCCCCG CTCGTTC-3' (reverse); β-actin, 5'-GACCTCTA TGCCAACACAG-3' (forward) and 5'-AGTACTTGCGCTCAGGAGGA-3’ (reverse); miR-30a-5p,5’-GAGCCAGGTTTCGTGGG CACGTGTGTTAT-3’ (forward) and 5'- ATAACACACGTGCCCACGAAACCTGGCTC-3' (reverse).

### 2.9. Colony formation assay

Approximately 1,000 cells were plated and cultured in DMEM medium for 7 d. Then, cells were stained with Wright’s stain, followed by staining with Sorensen phosphomolybdate buffer solution and Giemsa dye solution (9:1) for 10 min. Colonies were then counted under a microscope [28].

### 2.10. Transwell assay

HCC cells were transfected with shCASC7, pcCASC7, miR-30a-5p inhibitor and mimic or NC. Briefly, 3 × 10^5^ HCC cells were treated with 2.5 μg/mL mitomycin C to inhibit cell division for 24 h. After culturing, appropriate amount of trypsin digestion was added, cells were washed with PBS buffer and resuspend in serum-free medium. Then, cells were cultured in the basement with complete medium for 24 h. The superfluous medium was discarded. Cells were then fixed with polyformaldehyde (4%) for 30 min, followed by staining with crystal violet (0.1%) for 10 min. Ten visual fields were randomly selected, cells were observed, photographed, and counted [29].

### 2.11. Western blot analysis

Total proteins were extracted and separated by SDS-PAGE (10%). Protein samples were transferred onto PVDF membranes, which were blocked with primary antibodies of anti-KLF10 (1:1,000), TGF-b (1:1,000), p-SMAD3 (1:1,000), SMAD3 (1:1,000) and anti-GAPDH antibody (1:1,000) at 4°C overnight. Antibodies were purchased from Shifeng, Shanghai, China. Then anti-rabbit secondary antibody (1:1,000) was added to incubate the membrane [30]. After ECL (Sigma-Aldrich, USA) developed signals, Image J v1.46 software was used.

### 2.12. IHC staining

Liver cancer tissues were deparaffinized and heated after hydration. The sections were blocked with normal goat serum (10%) and then incubated with anti-Ki-67. The Envision™ ABC kit (Beinuo, Shanghai, China) was used for immunological detection. Finally, the Leica DM4000B/M microscope was used for observation [31].

### 2.13. Tumor xenograft models in nude mice

Male athymic nude mice were kept under standard conditions. Approximately 5 × 10^6^ HCC cells transfected with CASC7 lentiviral vector or miR-30a-5p inhibitor were separately subcutaneously inoculated into nude mice. All mice were divided to different groups. The tumor size was determined every week for 4 weeks. Mice were sacrificed and tumors were weighted.

### 2.14. Statistical methods

Data were presented as the mean ± stand deviation (SD). All statistical analyses were performed using the SPSS 13.0 software. Student’s t-test was used for comparisons. *p* value < 0.05 was considered as significant difference.

## 3. Results

In this study, we aimed to investigate whether and how CASC7 was involved in HCC progression. We found that CASC7 regulated the KLF10/TGF-β/SMAD3 axis via binding to miR-30a-5p, thereby promoting HCC cell progression.

### 3.1. CASC7 regulated the progression of HCC in vitro

**Figure 1.**
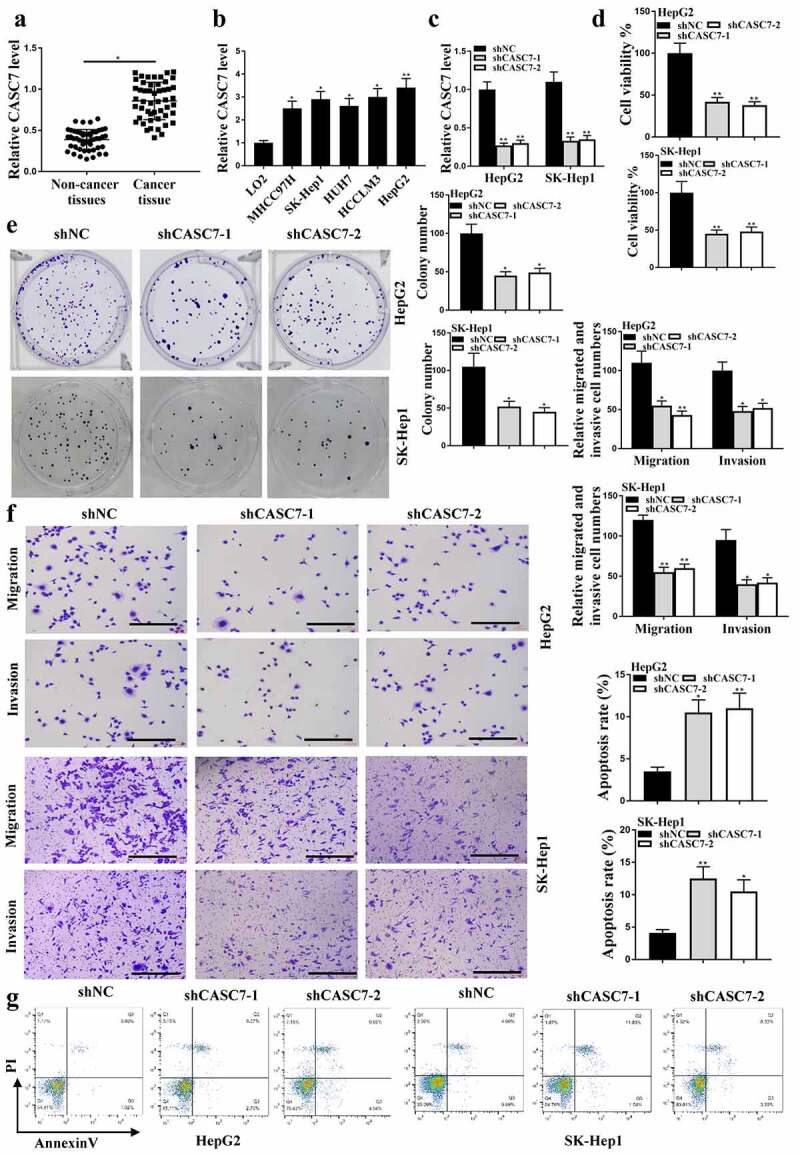
Effects of CASC7 on HCC tumor growth. (a) CASC7 expression in adjacent normal tissues and HCC tissues (n = 38). (b) CASC7 expression in HCC cell lines (c) CASC7 expression in HepG2 cells transfected with sh-NC, shCASC7-1 or shCASC7-2. (d) CCK8 determined Cell viability. (e and f) Colony formation, invasion and migration (100 um). (g) Cell apoptosis rate. ** *P* < 0.01, * *P*< 0.05; n= 3.

The expression levels of CASC7 in HCC tissues (n = 38) and HUH7, LO2, MHCC97H, SK-hep1, HepG2 cells were increased compared with that in adjacent normal tissues (Figure 1a) and HCCLM3 cells (*P* < 0.05, Figure 1b). The expression levels of CASC7 in HepG2 cells transfected with shCASC7-1 or shCASC7-2 were significantly reduced, indicating successful transfection (*P* < 0.05, Figure 1c). Colony formation (Figure 1e) and CCK-8 assay (Figure 1d) results showed that shCASC7-1 and shCASC7-2 significantly inhibited HepG2 cell viability and colony formation (*P* < 0.05). ShCASC7-1 and shCASC7-2 reduced HepG2 cell migration and invasion (figure 1f, p < 0.05). In addition, shCASC7-1 and shCASC7-2 significantly induced apoptosis of HepG2 cells (*P* < 0.05, Figure 1g). Altogether, these results suggested that CASC7 was involved in HCC progression *in vitro*.

### 3.2. MiR-30a-5p regulated HCC progression in vitro

**Figure 2.**
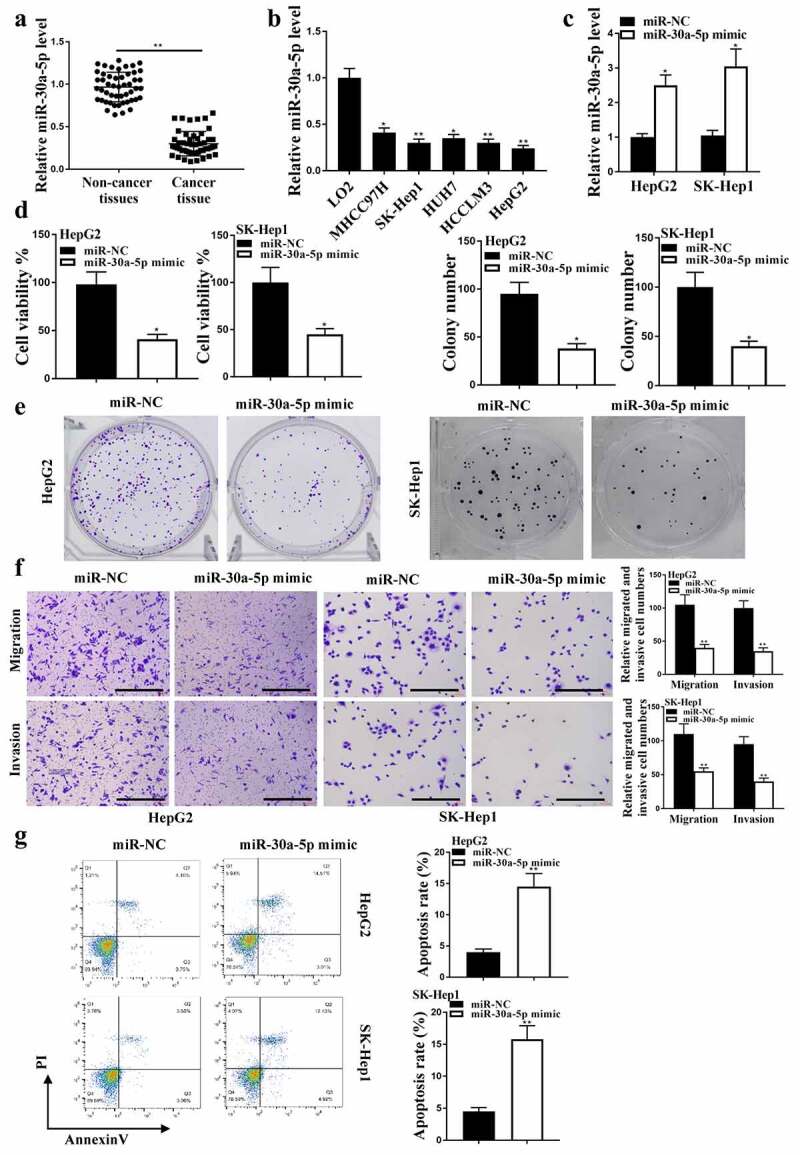
MiR-30a-5p in HCC proliferation. (a) In HCC and adjacent normal tissues (n = 38), miR-30a-5p expression was detected. (b) In HCC cell lines, miR-30a-5p expression was detected. (c) MiR-30a-5p expression in HepG2 and SK-Hep1 cells. (d) CCK8 assay for cell viability. (e and f) Colony formation, migration and invasion (100 um). (g) Cell apoptosis. ** *P*< 0.01, * *P* < 0.05; n = 3.

The expression levels of miR-30a-5p were reduced in HCC tissues (Figure 2a, p < 0.01) and HUH7, LO2, MHCC97H, SK-hep1, HepG2 cells (Figure 2b, p < 0.05) compared with that in adjacent normal tissues (n = 38) and HCCLM3 cells. The expression of miR-30a-5p was enhanced after transfection with miR-30a-5p mimic (*P* < 0.05, Figure 2c). CCK8 analysis (Figure 2d) and colony formation (Figure 2e) results showed that miR-30a-5p mimic reduced HepG2 cell viability and colony formation (*P* < 0.05), as well as HepG2 cell migration and invasion (figure 2f, p < 0.05), and induced HepG2 cell apoptosis (Figure 2g, p < 0.01). Taken together, these results suggested that miR-30a-5p regulated HCC progression *in vitro*.

## 4. MiR-30a-5p was a direct target of CASC7

**Figure 3.**
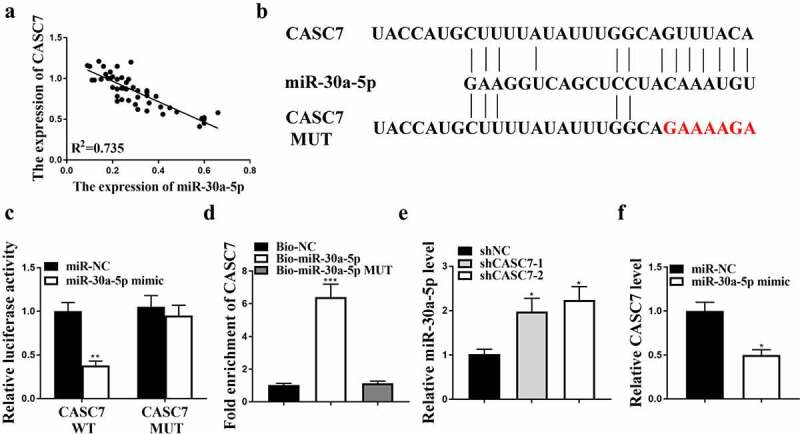
The interaction between miR-30a-5p and CASC7. (a) Correlation between CASC7 and miR-30a-5p. (b) The binding site between miR-30a-5p and CASC7. (c) Relative luciferase activity. (d) The interaction between miR-30a-5p and CASC7 evaluated by RNA pull-down. (e) MiR-30a-5p expression in HepG2 cells. (f) CASC7 expression in HepG2 cells. ** *P* < 0.01, * *P* < 0.05, n = 3.

The expression of CASC7 was inversely correlated with the expression of miR-30a-5p in HCC tissues (R^2^ = 0.76, Figure 3a). Starbase v2.0 and Tarbase v7.0 predicted multiple miRNAs that could bind with CASC7, including miR-30a-5p (Figure 3b). Moreover, the luciferase activity was decreased in HepG2 cells co-transfected miR-30a-5p mimic with CASC7-WT (*P* < 0.05), but not co-transfected CASC7-MUT with miR-30a-5p mimic (Figure 3c). In addition, the expression levels of CASC7 in HepG2 cells treated with miR-30a-5p probe were enhanced compared with HepG2 cells treated with NC probe (*P* < 0.05, Figure 3d). In contrast, the expression levels of miR-30a-5p in the shCASC7-1 and shCASC7-2 groups were enhanced (Figure 3e, p < 0.05). The expression levels of CASC7 were reduced after overexpression of miR-30a-5p (*P* < 0.05, Figure. 3f). Taken together, these results suggested that miR-30a-5p was a direct target of CASC7.

## 5. MiR-30a-5p mediated the effect of CASC7 on HCC cell proliferation

**Figure 4.**
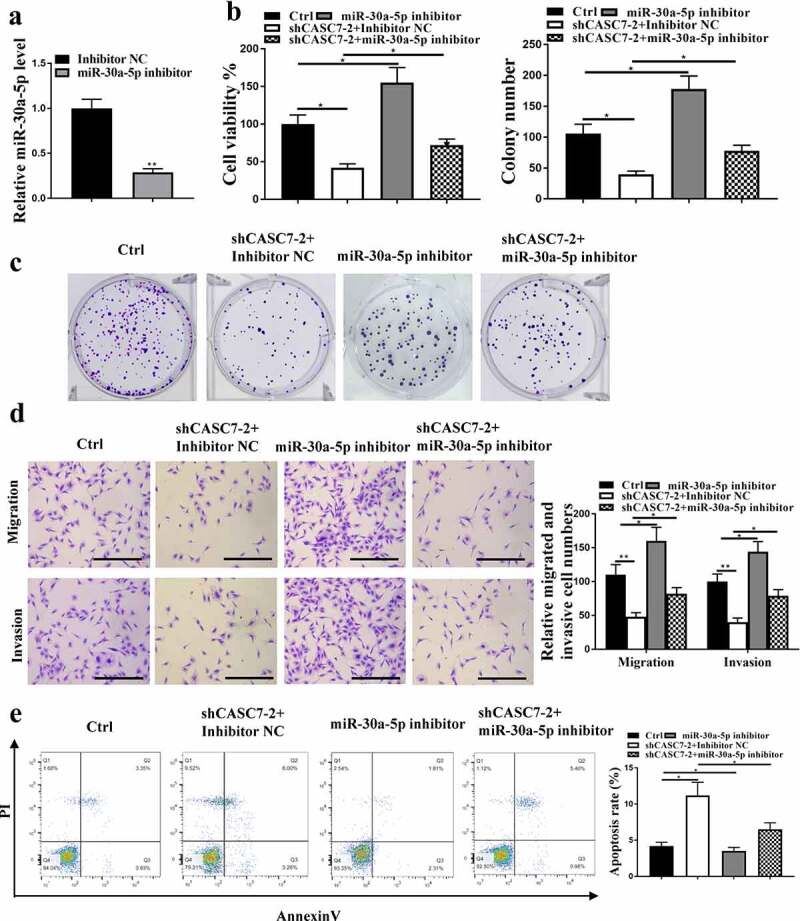
The effect of CASC7 on HCC proliferation mediated by miR-30a-5p. (a) miR-30a-5p expression in HepG2 cells. (B, C and D) migration and invasion (100 um), colony formation, cell viability. (e) Cell apoptosis rate. ** *P* < 0.01, * *P* < 0.05, n = 3.

The expression levels of miR-30a-5p were reduced after inhibition of miR-30a-5p (p < 0.05, Figure 4a). ShCASC7 reduced HCC cell proliferation (Figures 4b and 4c, p < 0.05), which was reversed by miR-30a-5p inhibition. Moreover, shCASC7 significantly inhibited HCC cell invasion and migration, which was also reversed by inhibition of miR-30a-5p (Figure 4d, p < 0.05). Moreover, shCASC7 induced HCC cell apoptosis, which was reversed by miR-30a-5p inhibitor, too (Figure 4e, p < 0.05). Altogether, these results suggest that miR-30a-5p mediated the function of CASC7 in HCC cell proliferation.

### 5.1. KLF10 was a direct target of miR-30a-5p

**Figure 5.**
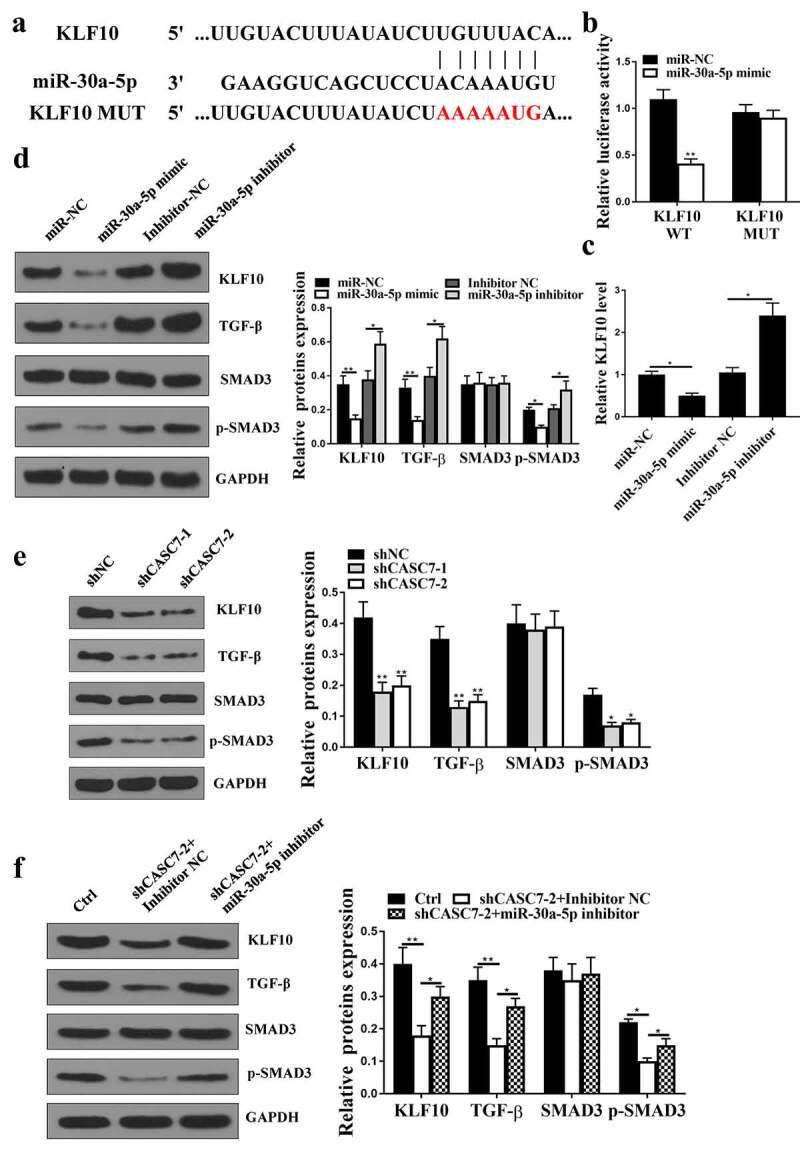
KLF10 was predicted. (a) The binding site between miR-30a-5p and KLF10. (b) Relationship between KLF10 and miR-30a-5p evaluated by relative luciferase activity assessment. (c) KLF10 expression in HepG2 cells. (d) KLF10, SMAD3, p-SMAD3, TGF-β and protein expression in HepG2 cells. (e) KLF10, SMAD3, p-SMAD3, TGF-β protein expression in HepG2 cells treated with shCASC7-1 and shCASC7-2. (f) KLF10, SMAD3, p-SMAD3, TGF-β protein expression in HepG2 cells treated with shCASC7 + Inhibitor NC and shCASC7 + miR-30a-5p inhibitor. ** *P* < 0.01, * *P* < 0.05, n = 3.

Starbase v2.0 predicted that KLF10 was a potential target of miR-30a-5p (Figure 5a). Co-transfection of KLF10-WT with miR-30a-5p mimic (Figure 5b, p < 0.05) reduced luciferase activity in cells, but not that of the co-transfection of KLF10-MUT with miR-30a-5p mimic (Figure 5b, p > 0.05) in cells. In addition, the expression levels of KLF10 were reduced at both mRNA (Figure 5c) and protein (Figure 5d) levels in miR-30a-5p mimic group (*P* < 0.05), but increased in miR-30a-5p inhibitor group (*P* < 0.05) compared with that in the NC group. The expression levels of p-SMAD3 and TGF-β were decreased in miR-30a-5p mimic group (Figure 5d, p < 0.05), but enhanced in miR-30a-5p inhibitor group (Figure 5d, p < 0.05). Knockdown of CASC7 significantly reduced the expression levels of KLF10, p-SMAD3 and TGF-β (Figure. 5e, p < 0.05), which was reversed by miR-30a-5p inhibition (p < 0.05, Figure 5f). Altogether, these results suggested that KLF10 was a direct target of miR-30a-5p.

### 5.2. Down-regulation of CASC7 restrained HCC progression in vivo

**Figure 6.**
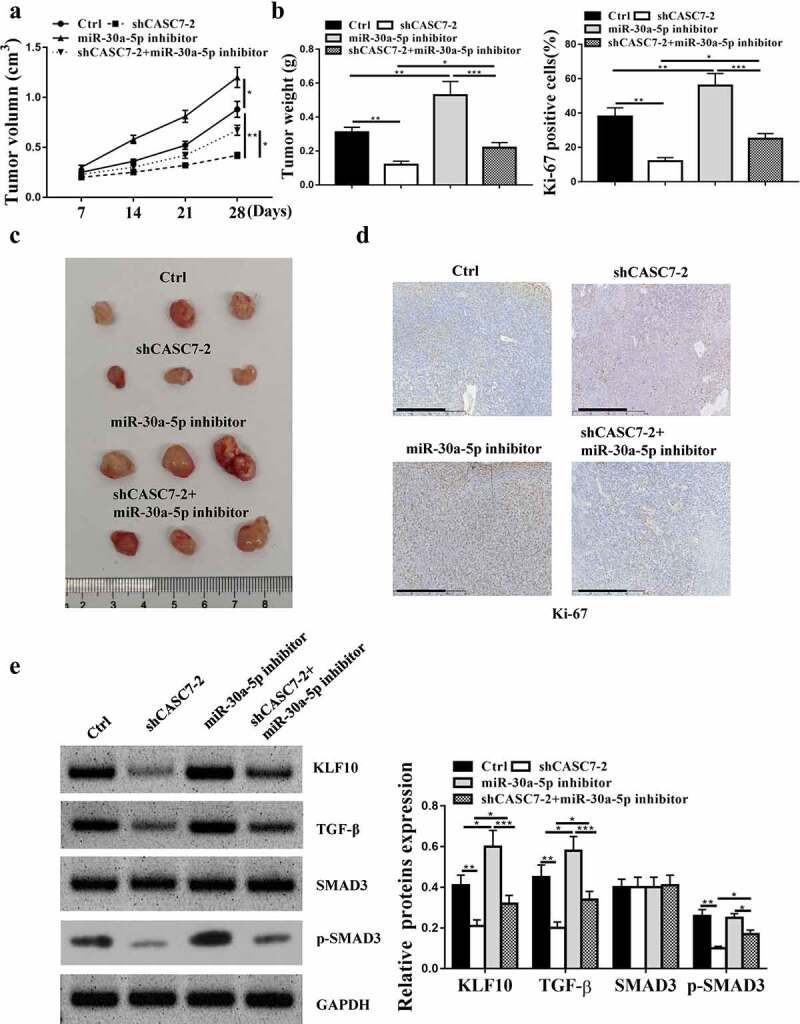
CASC7 in HCC tumor cell growth *in vivo*. (a and b) Tumor volume and weight was detected. (c) Three groups of subcutaneous tumors’images. (d and e) Ki-67 staining (100 um), KLF10, TGF-β and SMAD3 protein expression in tumor tissues. ** *P* < 0.01, * *P* < 0.05, n = 4.

It was found that inhibition of miR-30a-5p enhanced the tumor volume and weight, and knockdown of CASC7-2 reduced the tumor volume and weight (Figure 6a-6c, p < 0.05), which was attenuated by inhibition of miR-30a-5p. Immunohistochemical results showed that Ki67 positive cell number was increased in miR-30a-5p inhibitor group and decreased in shCASC7-2 group, which was attenuated by miR-30a-5p inhibitor (Figure 6d). ShCASC7-2 reduced the expression levels of KLF10, TGF-β and p-SMAD3 (*P* < 0.05), which was attenuated by miR-30a-5p inhibitor (*P* < 0.05, Figure 6e). Compared with the Control, inhibitor-NC and sh-NC group, shCASC7-2 significantly reduced the expression levels of KLF10, p-SMAD3 and TGF-β, which was attenuated by miR-30a-5p inhibition (supplementary Figure 1, *P* < 0.05). Altogether, these results suggested that knockdown of CASC7 inhibited HCC progression *in vivo*.

## 6. Discussion

Even with encouraging progress that has been made in the understanding of the molecular mechanisms of liver cancer development, in advanced liver cancer patients, the prognosis remains unfavorable [32]. It is known that altered expression of lncRNAs are closely related to the occurrence and development of liver cancer [33,34]. Here, we showed that the expression levels of CASC7 were increased in HCC tissues and cells. Importantly, CASC7 promoted HCC tumor malignant processes and tumor growth in vivo, suggesting that CASC7 plays an oncogenic role in HCC.

LncRNAs were shown to be involved in pathological processes of human disease [35]. In tumor development process, lncRNAs exert their functions as pro-tumor factors or suppressors in tumors through various mechanisms, such as regulating the expression of neighboring genes or affecting the expression of related genes [36]. Studies have identified many abnormally expressed lncRNAs in HCC, which are involved in HCC prognosis, metastasis and occurrence [37]. For example, the highly upregulated expression of ANRIL is closely related to HCC poor prognosis [38] and induces HCC cell invasion and proliferation [39]. In a variety of malignancies, CASC7 was found to be abnormally expressed. For example, the expression levels of CASC7 in colorectal cancer are significantly decreased, while overexpression of CASC7 can inhibit the survival of colorectal cancer cells [40]. In glioma tissues and cell lines, CASC7 is down-regulated and involved in glioma cell proliferation and apoptosis [13]. Moreover, it was reported that CASC7 suppresses malignant behaviors of breast cancer by regulating the miR-21-5p/FASLG axis [27]. Here, we demonstrated that the expression levels of CASC7 were significantly increased in HCC. Moreover, knockdown of CASC7 inhibited HCC cell invasion, migration, and proliferation and induced HCC cell apoptosis. These suggested that CASC7 acts as an oncogene in the occurrence of HCC. Our findings further enriched our understanding of CASC7 in different types of tumor.

Studies have shown that lncRNAs have sponge adsorption on miRNAs, and thus participate in tumorigenesis [41]. For example, lncRNA DLEU2 sponges miR-30a-5p to accelerate non-small cell lung tumorigenesis and invasion [42]. LncRNA MALAT1 sponges miR-30a-5p to regulate vimentin expression in HCC [43]. Here, miR-30a-5p was found to be a target gene of CASC7. The expression levels of miR-30a-5p were reduced in HCC and reversely correlated with the expression levels of CASC7. In addition, overexpression of miR-30a-5p reduced HCC cell migration, invasion and proliferation and induced apoptosis of HCC cells, and it could reverse the effects of shCASC7 on HCC tumor growth and cell apoptosis, invasion, migration, proliferation. These suggest that miR-29a-3p mediated the function of CASC7 in HCC.

KLF10 is a transcription factor containing a zinc finger protein domain, and TGF-β is an early responsive gene [44]. It has been reported that the TGF-β/SMAD3 axis is closely related to HCC occurrence [45]. Here, KLF10 was a target gene of miR-30a-5p. The expression levels of KLF10, p-SMAD3 and TGF-β were reduced after overexpression of miR-30a-5p. ShCASC71 could reduce the expression levels of KLF10, p-SMAD3 and TGF-β, which was reversed by miR-30a-5p inhibitor. These indicate that CASC7 regulates KLF10/TGF-β/SMAD3 through miR-30a-5p to promote HCC proliferation. In future, we will further investigate the roles of SMAD2 and SMAD4 in HCC proliferation.

## Supplementary Material

Supplemental Material

## Data Availability

The analyzed data sets generated during the study are available from the corresponding author on reasonable request.
