# Supplementary figures and images for: Inhibition of long noncoding RNA cancer susceptibility candidate 7 attenuates hepatocellular carcinoma development by targeting microRNA-30a-5p
Source: Bioengineered. 2022 Apr 29;13(4):11296–308. doi: 10.1080/21655979.2022.2068289 (PMC9208517; doi:10.1080/21655979.2022.2068289)

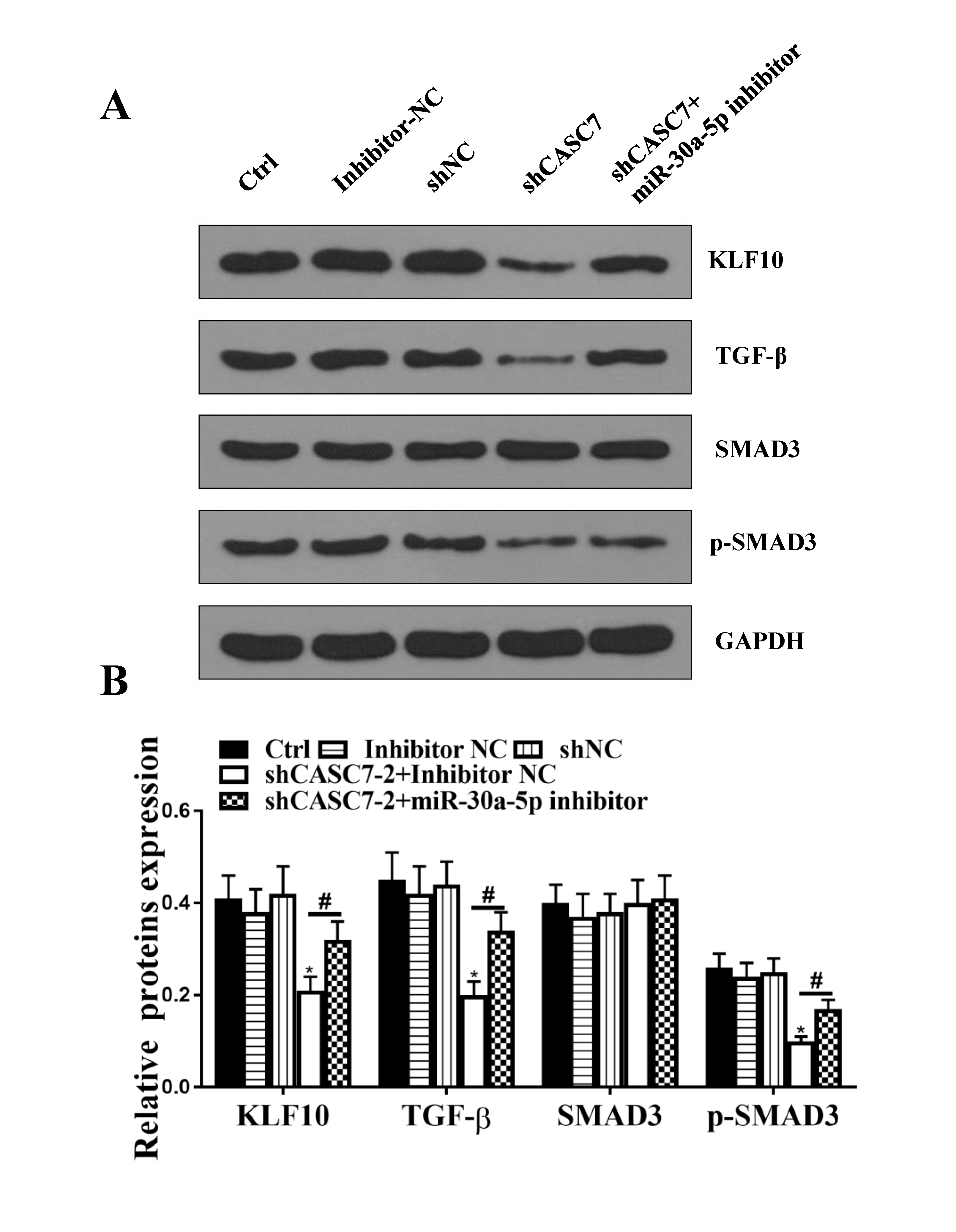

Supplement: Supplemental Material [file KBIE_A_2068289_SM0924.zip › supplementary/Supplementary.tiff]

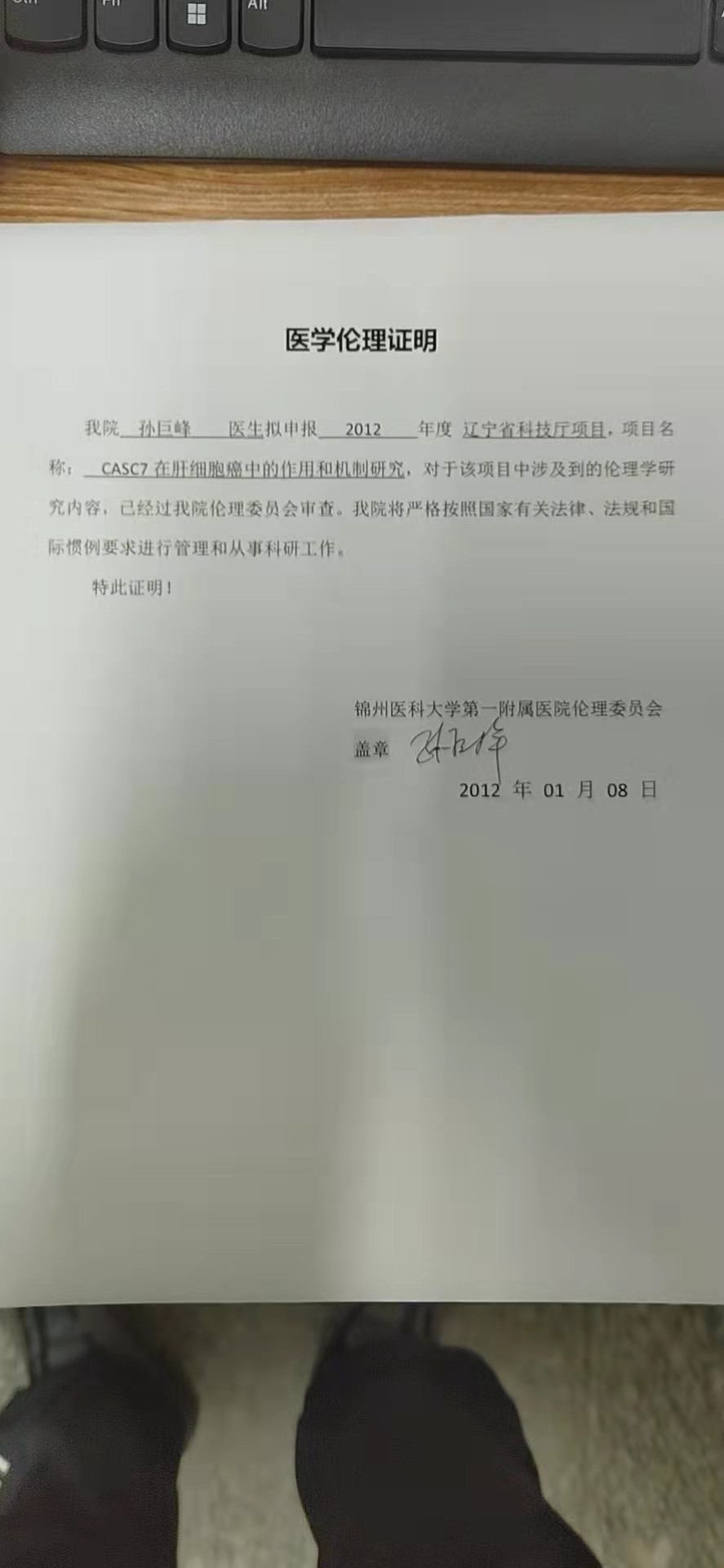

Supplement: Supplemental Material [file KBIE_A_2068289_SM0924.zip › supplementary/_.jpg]
